# Case Study of Airborne Pathogen Dispersion Patterns in Emergency Departments with Different Ventilation and Partition Conditions

**DOI:** 10.3390/ijerph15030510

**Published:** 2018-03-13

**Authors:** Chang Heon Cheong, Seonhye Lee

**Affiliations:** 1 Department of Architectural Engineering, Gyeongnam National University of Science and Technology, Jinju 52725, Korea; 2 Department of Nursing, Gyeongnam National University of Science and Technology, Jinju 52725, Korea

**Keywords:** airborne pathogen, emergency department, CFD simulation, ventilation, partition

## Abstract

The prevention of airborne infections in emergency departments is a very important issue. This study investigated the effects of architectural features on airborne pathogen dispersion in emergency departments by using a CFD (computational fluid dynamics) simulation tool. The study included three architectural features as the major variables: increased ventilation rate, inlet and outlet diffuser positions, and partitions between beds. The most effective method for preventing pathogen dispersion and reducing the pathogen concentration was found to be increasing the ventilation rate. Installing partitions between the beds and changing the ventilation system’s inlet and outlet diffuser positions contributed only minimally to reducing the concentration of airborne pathogens.

## 1. Introduction

### 1.1. Background

Emergency departments (ED) are visited by patients with various diseases, and thus, are vulnerable to the dispersion of airborne pathogens [1,2,3]. In particular, if the hospital does not have isolation rooms [4,5,6] for the treatment of patients carrying airborne pathogens or has more respiratory patients visit the hospital than the number of beds available in the isolation rooms, it is possible that patients with respiratory problems will be assigned to normal beds in the ED. In this situation, it is anticipated that the possibility of airborne pathogen dispersion in the ED increases. Airborne pathogens are particles that are 5 μm or smaller (aerosol) [7]. As they travel in the air, their dispersion to nearby areas is possible, depending on the indoor air conditions. Controlling the indoor air patterns, installing partitions between units to prevent airborne pathogen dispersion, and installing UV lamps to sterilize the pathogens can be considered as alternatives to prevent the dispersion of airborne pathogens inside EDs. Partitions are used in target ED in hospitals as a means to control the dispersion of airborne pathogens [8].

Among these options, we focused on the effects of arrangement of diffusers, the installation of partitions between beds, and increased ventilation rate affect the airflow patterns in an ED. Each of these factors was applied to the bed areas of an ED, and the evolution of airborne pathogen dispersion was analyzed through a CFD (Computational Fluid Dynamics) simulation.

### 1.2. Purpose of the Study

As mentioned previously, the objectives of the research were to investigate the effects of the locations of ventilation system diffusers, the presence of partitions between beds, and increased ventilation rate on the dispersion of airborne pathogens. Therefore, the following scenarios were analyzed.

First, the ventilation around the beds in the ED was increased from the regulated ventilation level of three air change per hour (ACH) to the ventilation level of six ACH, which is required for a negative-pressure isolation chamber. Isolation rooms for patients with airborne pathogens require a minimum ventilation rate of 6–12 ACH [5,6]. Then, the effect on the pathogen dispersion control was analyzed.

Second, the location of diffusers was changed around the beds in the ED, and the consequential changes observed in the pathogen dispersion patterns were analyzed. In this case, the conditions were largely in one of two categories: supplying and exhausting the air over the respiratory system of a patient. An alternative was designed by considering the general design of inlets and outlets for negative pressure chambers, in which the air is exhausted at the restroom side or around the respiratory system of the patient.

Last, an additional partition was installed between the ED beds, and the degree of change observed in the pathogen dispersion patterns was analyzed. This approach is thought to be an alternative that can prevent the dispersion of airborne pathogens to adjacent beds.

In South Korea, generally, ED is designed with an open plan and the airborne pathogen can freely migrate from infected patient to any place in ED. The conventional fabric partition or curtains between beds easily pass the airborne pathogens smaller than 5 μm. The impermeable partitions that have benefits such as easy installation and cheap price are thought to be useful to prevent the air pathogen transfer from a bed to another. However, airflow pattern and ventilation rate can influence the effects of the partition regarding airborne pathogen dispersion. This study investigates the combined effects of ventilation rate, inlet/outlet location and existence of partition in ED on airborne pathogen dispersion and consequent infection risk. The results of this study are expected to provide an effective architectural design guide for ED to prevent airborne infections.

## 2. Methods

### 2.1. Selected Spaces for the Study

**Figure 1:**
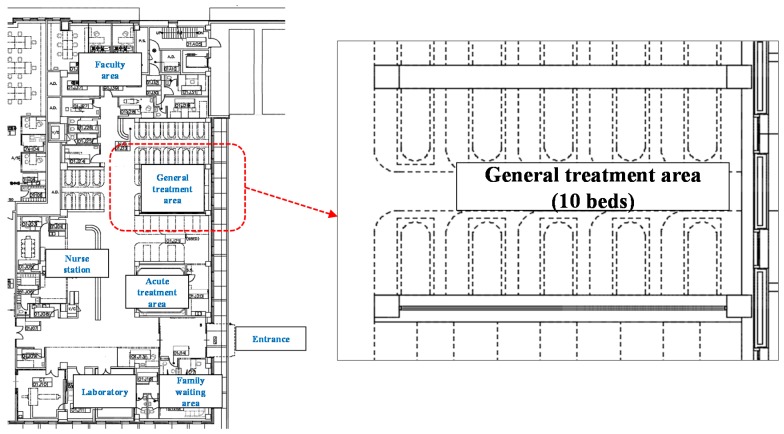
Target area in the ED (emergency department).

The subject for analysis was an ED based in Seoul. The ED was consisted of faculty area, nurse station, laboratory, family waiting area, acute treatment area and general treatment area. The general treatment area where the emergency beds were located in the ED was selected as the analysis model. Figure 1 illustrates target area with ten beds without partitions. In the actual conditions, the space was open to some other areas in the department. The conditions concerning the surrounding areas, which cannot be specified, were excluded and the subject space was independently modeled in the analysis.

### 2.2. Simulation Tool

A CFD analysis program, Star-CCM+ v10, was used to achieve the research objectives. Star-CCM+ is a CFD simulation tool to fluid dynamics, particle/gas flow and heat transfer in a controlled volume. This software provides lots of physics models and analysis tools for the investigation. The airflow dispersion patterns and the airborne pathogen density distribution were analyzed in the study to estimate airborne infection risk. The subjects of the CFD analysis were the airflow and ventilation conditions of the bed area in the ED, and variation in the pathogen dispersion trend with the installation of partitions between the beds. For the Star-CCM modeling, only the indoor air area was modeled as a solid object in AutoCAD 2015 (computer-aided design, Autodesk, San Rafael, CA, USA), and imported to Star-CCM+. A polyhedral mesh was selected for the analysis model, and a prism layer mesh was applied. For the equations governing the flow field of the three-dimensional model, the continuity equation, momentum equation, and equations related to the degree of turbulence were applied. For the turbulence analysis, the Realizable *k-ε* Turbulence model was used. The segregated flow model was selected for the fluid model.

### 2.3. Modeling Condition

**Figure 2:**
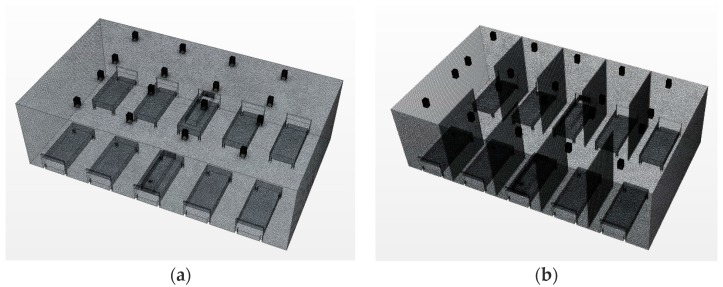
Geometric features of the simulation models and space meshing conditions. (**a**) Base model (without partitions between the beds); (**b**) Type 1 model (with partitions between the beds).

In this research, two analysis models were established based on the installation of partitions. Figure 2 shows the image of each simulation model with and without partition. The images of meshing conditions are also depicted in Figure 2. The mesh and number of cells for each condition, and the boundary conditions are listed in Table 1 and Table 2, respectively. The air supply and exhaust were in the upper area shown in Figure 2. All patients were lying in the beds with their heads near the wall. According to the literature by Sung (2015), the minimum ventilation requirements for hospitals in South Korea, as defined by current regulations, is approximately 2.4 ACH. Based on this, the baseline ventilation condition without airborne pathogen control was set at 3 ACH in this study. The entire air supply was assumed to come from the outside [9]. The ventilation condition for pathogen control was set at 6 ACH. This is the minimum value in the range of 6 ACH–12 ACH, which is the required ventilation rate inside negative pressure units suggested by the “Plan for extended installation of negative pressure units at nationally designated isolation hospitals” published by the Korea Centers for Disease Control & Prevention in 2015 [10].

**Figure 3:**
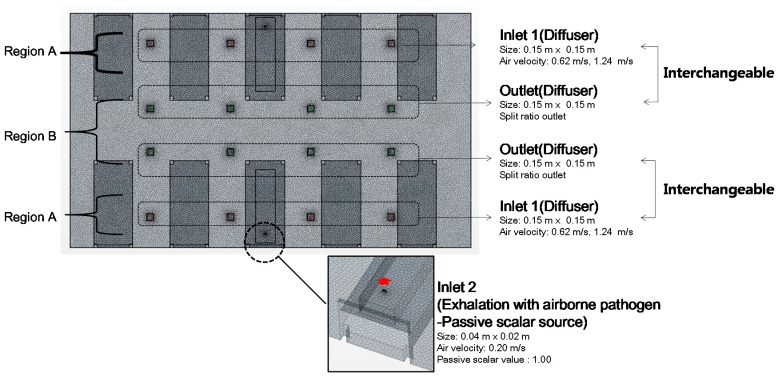
Inlet/outlet locations of the model in the analysis.

**Figure 4:**
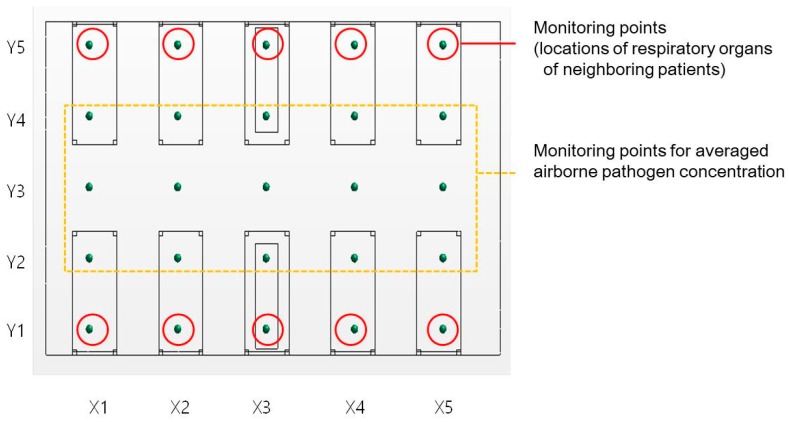
Monitoring points for the concentrations of the airborne pathogen.

The numbers of inlets and outlets, as shown in Figure 2 and Figure 3, were slightly different in the base model and the Type 1 model. This was because the diffusers were placed in the spaces between the beds in the base model so that the ventilation would not occur directly in the face of the patient. The Type 1 model applied walls between each bed, and the ventilator supply was placed at every bed. The inlet condition for the Star-CCM+ simulation model was set to the velocity inlet, and the outlet condition was set as a split ratio outlet. The buoyancy effect was considered, and the detailed thermal conditions of the simulation models are shown in Table 1. Assuming that the patient breathed 18 times/min, had a breathing capacity of 500 mL (about 9 L/min), and their mouth measured 0.04 m × 0.02 m, the average air velocity of exhalation was calculated to be approximately 0.19 m/s. Based on this, therefore, the average exhalation velocity was set in the model to be 0.2 m/s (red arrow in Figure 3.). Patients were assumed to be lying in the center of their beds. The exhalation was emitted from one patient, and the analysis was conducted accordingly. A passive scalar was selected to represent the pathogen included in the exhalation, and the concentration was 1.0. According to previous study [11], the passive scalar method is also a good approach of small particles bioaerosols. The airborne pathogen dispersion pattern resulting from the exhalation, mixing of airflows, and turbulence patterns was visualized for analysis. Figure 4 shows the data measuring points for the analysis of pathogen concentration. The points were set at a height of 1.0 m, in consideration of the location of the respiratory system of a patient lying in a bed. The meshing conditions of the base (2,405,265 cells) and Type 1 (2,691,618 cells) models are provided in Table 2. The black boxes above the beds are inlets and outlets of the ventilator. The bulge of the bed is 2.0 m × 0.9 m × 0.5 m. Human body is expressed in Figure 2 and mouth is modeled top of the human body(0.04m × 0.02 m, 0.7 m height).

### 2.4. Simulation Cases

Simulation cases for analysis were established, as shown in Table 3, to conduct a case study that analyzed the range of airborne pathogen dispersion according to the location of diffusers in the bed area of the ED, the ventilation rate, and the installation of partitions between beds.

Case 1 is the condition that best represents the usual bed conditions of existing EDs. The ventilation rate was 3 ACH, per regulation, with no partitions between the beds. Fabric curtains are usually installed between the beds, but it was assumed that the curtains were left open, as in situations where undressing or exposing the body was not necessary.

The inlets were around the periphery of the ED (Region A), and the outlets were in the central area (Region B). The inlet was located near the side of the patient’s respiratory system. The conditions of Case 2 were the same as in Case 1, only with the locations of the inlet and outlet switched, so that the outlet was located over the patient’s respiratory system. Cases 3 and 4 were the same as Cases 1 and 2, respectively, with the ventilation rate increased to 6 ACH. Cases 5–8 had the same conditions as Cases 1–4, except for having partitions in place between the beds. As in the previous explanation of the analysis model, the total number of inlets and outlets was increased, and the air velocity at the inlets decreased accordingly with the installation of a diffuser for every bed.

### 2.5. Wells–Riley Equation

The Wells–Riley model is a method of expressing the probability of air-borne infection using the concentration of a steady-state infection source and thread ventilation [12,13,14,15,16]. Equation (1) shows the expression of the Wells–Riley model. Equation (2) shows the modified Wells–Riley model for infection risk by spatial area.

(1) $$P_{I}=\frac{C}{S}=1-e^{-\frac{Iqpt}{Q}}$$

where:

*P_I_*: Airborne infection probability of a susceptible person;
*C*: Number of infection cases;
*I*: Number of infectors;
*p*: Breathing rate of a susceptible person; and
*q*: Quantum generation rate by an infector.

(2) $$P_{I}=1-e^{-pNt}$$

where *N*: Expected number of quanta.

### 2.6. Exposure Condition

The quanta generation rate of influenza was set to 515 particles/h [17,18]. The existing literature suggests that the quanta generation rate of influenza is 15–500 particles/h [19].

**Figure 5:**
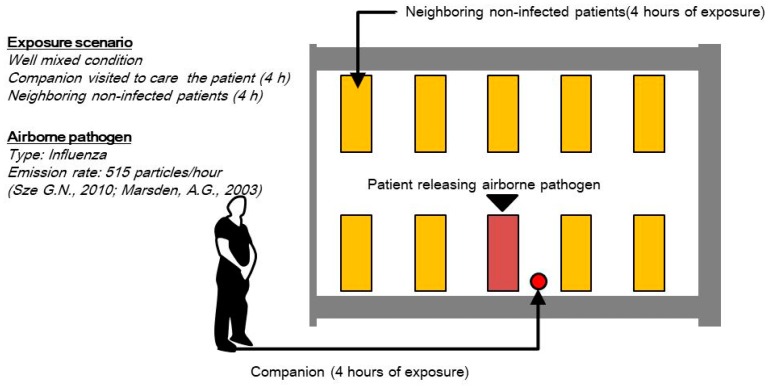
Quanta generation scenario.

In South Korea, after the Middle East respiratory syndrome (MERS) outbreak in 2015, only one patient companion can stay in the ED and take care of the patient near their bedside. Other patient companions must wait in the waiting room. The duration of a patient’s stay in the emergency room depends on the patient’s condition, but in the worst case, a patient may stay for two or three days. However, in this study, we assumed that the condition is treated for 4 h:2 h for blood tests and 2 h of other medical treatments by hospital members’ interview. The quanta generation scenario is depicted in Figure 5. Airborne pathogen concentration of each monitoring point is used to calculate airborne infection risk of neighboring non-infected patient. Airborne pathogen concentration at X3-Y1 is used to evaluate companions’ airborne infection risk.

## 3. Results

### 3.1. Pathogen Concentrations at Monitoring Points

**Figure 6:**
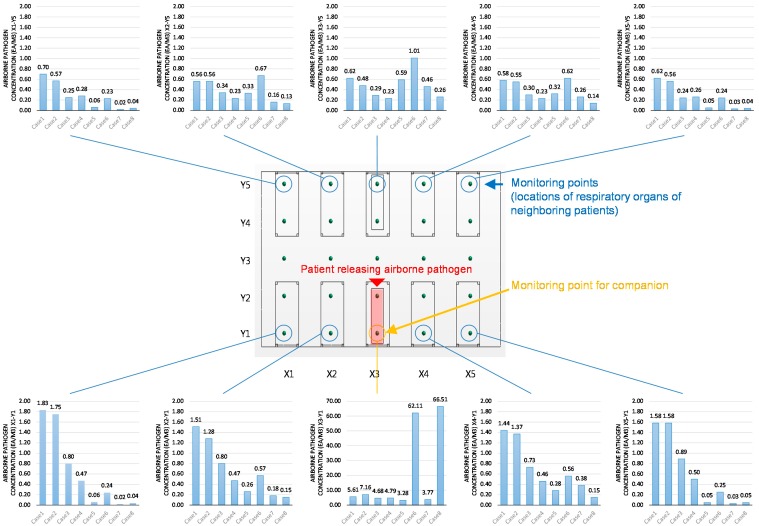
Average pathogen concentration for all cases.

**Figure 7:**
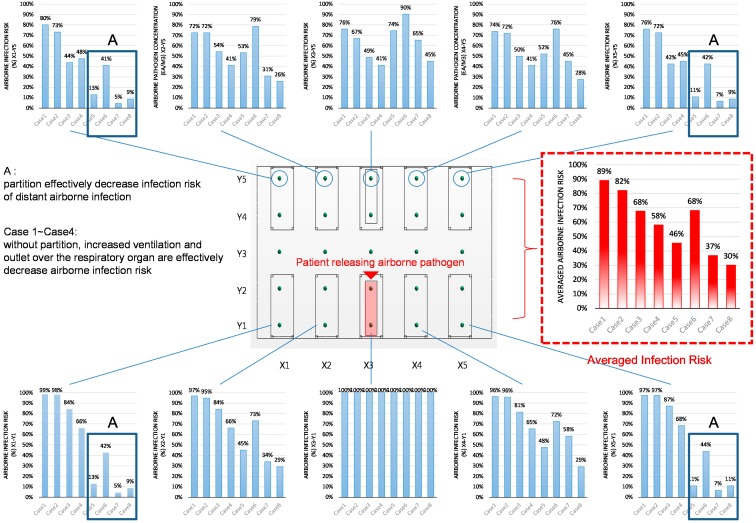
Airborne infection risk for all cases (4 h of exposure).

Figure 6 and Figure 7 depict the distribution of the pathogen concentration and infection risk at the measuring points shown in Figure 4.

Figure 6 shows the average concentration at all measuring points together with the beds of the patient with a respiratory problem who generated the pathogen (X3-Y1 and X3-Y2), the beds opposite to the pathogen source patient (X3-Y4 and X3-Y5), and the average pathogen concentration at the rest of the beds.

According to Figure 6, there was a large difference between the pathogen concentrations at the pathogen source bed and that at the rest of beds. First, the reduction of pathogen concentration with increased ventilation rate is well demonstrated in all cases analyzed (Cases 1 and 2 vs. Cases 3 and 4 and Cases 5 and 6 vs. Cases 7 and 8).

The effect of switching the ventilation outlet from Region B to Region A varied depending on the existence of partitions. In the condition without partitions, there was minimal effect on the average concentration at the periphery, and the pathogen concentration at the pathogen source bed showed a slight increase (X3). However, the change of outlet location (from Region B to Region A) reduced the dispersion of the pathogen to the adjacent beds (X1, X2, X4, and X5).

Placing a partition between the beds when the outlet location was at Region B did slightly decrease the concentration at the pathogen source beds (X3-Y1) as compared to the condition without partitions. In the case of the condition with the partitions, switching the outlet location from Region B to Region A increased the concentration at the pathogen source bed. In addition, comparing Cases 5 and 6, changing the outlet location from Region B to Region A when partitions were between the beds contributed to a significant increase in the pathogen concentration at the adjacent bed (X1–X5). The pathogen concentration at X2-Y5 and X3-Y5 in Case 6 exceeded the corresponding values in Case 1 and 2. However, installing partitions between the beds (Case 6 vs. Cases 1 and 2) was confirmed to slightly reduce the average concentration levels at the rest of the beds on the periphery.

Figure 7 shows the expected infection risk at each measuring point. The infection risk reflects the tendencies of the pathogen concentrations in each case; a higher pathogen concentration means a higher infection risk. Increasing the ventilation rate is always desirable for the reduction of infection risk. Figure 7 illustrates that installing partitions contributed to a reduced infection risk in the periphery (A) and a clear reduction in the average infection risk at the increased ventilation rate of 6 ACH. However, the increased infection risk at the bed adjacent to the airborne pathogen generation source should not be neglected in the cases with partitions between the beds.

Estimated number of airborne pathogens and airborne infection risk at each monitoring point is demonstrated in Appendix A.

### 3.2. Airborne-Pathogen Concentration in the Cases without Partitions

#### 3.2.1. Changing the Diffuser Locations

**Figure 8:**
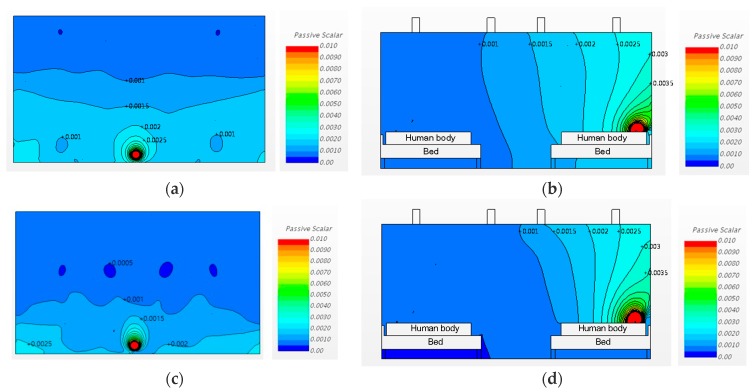
Airflow patterns and pathogen concentrations for Cases 1 and 2: (**a**) Case 1, plan view (h = 1.0 m); (**b**) Case 1, sectional view; (**c**) Case 2, plan view (h = 1.0 m); (**d**) Case 2, sectional view.

Case 1 and 2 had the same ventilation rate of 3 ACH, but the ventilation system inlet and outlet locations were switched to compare the consequent pathogen dispersion patterns. Figure 8 shows the analysis results for Cases 1 and 2 in plan (at 1.0 m height) and sectional (X3) views. Figure 8a,b are the analysis results, in plan and sectional views, respectively, for Case 1, in which the air was supplied from the periphery and exhausted at the center of the room. Figure 8c,d show the results of a central air supply and peripheral exhaust, in other words, exhaust over the respiratory systems of patients, as in the conditions of Case 2. White parts of Figure 8b,d are the bed and human body section.

In terms of the pathogen concentration distribution, the pathogen normally dispersed towards the adjacent beds, in both Cases 1 and 2. The concentration in the area near the bed of the patient who released the airborne pathogen was slightly higher in Case 1, where the air was supplied from the periphery (Region A) that was above the patients’ respiratory systems. The same pattern can be observed in Figure 8a,c, which show the sectional views. These figures show that, in both Cases 1 and 2, the pathogen dispersed towards the beds on the opposite sides. The pathogen concentration at the hallway side was higher in Case 1 than in Case 2. It is therefore anticipated that the probability of infection for the people in the hallway and the patient on the other side would be greater in Case 1 than in Case 2.

#### 3.2.2. Changing the Diffuser Locations and Increasing the Ventilation Rate

**Figure 9:**
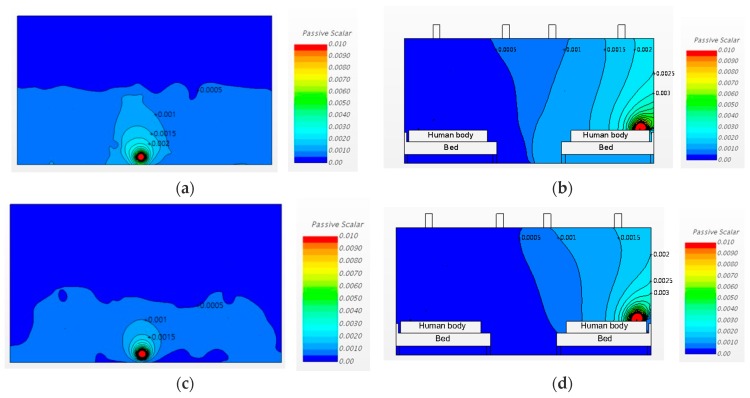
Airflow patterns and pathogen concentrations for Cases 3 and 4: (**a**) Case 3, plan view (h = 1.0 m); (**b**) Case 3, sectional view; (**c**) Case 4, plan view (h = 1.0 m); (**d**) Case 4, sectional view.

Cases 3 and 4 are the same as Cases 1 and 2, respectively, except for the ventilation rate. The ventilation rates for Cases 3 and 4 were increased to 6 ACH. The analysis results for Cases 3 and 4 are depicted in Figure 9. The concentrations of airborne pathogens in Cases 3 and 4 were clearly lowered when the ventilation rate was 6 ACH, as compared to 3 ACH. In other words, pathogen control by increasing the ventilation rate would have a greater effect on the pathogen concentration than changing the ventilation inlet and outlet locations would.

### 3.3. Airborne Pathogen Concentrations in Cases with Partitions

#### 3.3.1. Changing the Diffuser Locations

Cases 5 and 6 have the same ventilation conditions as Cases 1 and 2, respectively, with partitions added between the beds. The upper and lower parts of these partitioning walls were fixed to the ceiling and the floor, thus spatially separating the pathogen source bed from the adjacent beds. The front side remained open. This partitioning format not only controlled the dispersion of airborne pathogens from the source bed to the neighboring beds but also limited the visual field of the medical personnel.

**Figure 10:**
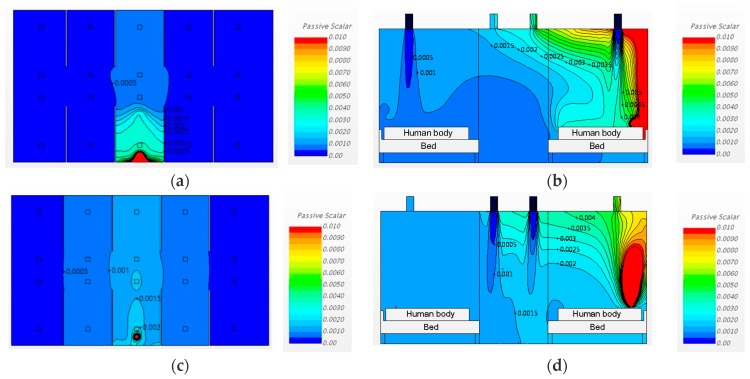
Airflow patterns and pathogen concentrations for Cases 5 and 6: (**a**) Case 5, plan view (h = 1.0 m); (**b**) Case 5, sectional view; (**c**) Case 6, plan view (h = 1.0 m); (**d**) Case 6, sectional view.

Figure 10a,c show, in plan view, the pathogen dispersion for the conditions of changing the inlet locations at the ventilation rate of 3 ACH. In Case 5, the air was supplied over the patient’s respiratory system (Region A), and the air supply pattern in sectional view was as shown in Figure 10b. The pathogen concentration for Case 5 in plan view is shown in Figure 10a. In Case 6, the air was supplied from the hallway side (Region B), resulting in concentrations in sectional view as shown in Figure 10d, and plan view as shown in Figure 10c.

Considering Figure 10, it is evident that the partition delayed the airborne pathogen dispersion to the adjacent beds. When the ventilation system inlets were installed over the patients’ respiratory systems (Region A), the airflow into the inlet acted like an air-curtain and prevented the airborne pathogen dispersion from the emitting source to other places, and the outlet at the hallway provided a secondary barrier (Case 5). Interestingly, in Case 6, with a 3 ACH ventilation rate, the airborne pathogens were not sufficiently exhausted at the outlet above the patients’ respiratory organs. The rest of the airborne pathogen moved to the hallway, and the airflow from the inlet installed in the hallway accelerated the dispersion of the airborne pathogen to the adjacent beds. As a result of these dispersion patterns, installing partitions caused airborne pathogen concentration to increase at X2 and X4. However, installing partitions contributed to a decrease in the airborne pathogen concentrations at X1 and X5. In Case 6, the pathogen concentration in the pathogen source bed greatly increased compared to the other cases, which could increase the infection risk for medical staff working in the source bed area.

#### 3.3.2. Changing the Diffuser Locations and Increasing the Ventilation Rate

**Figure 11:**
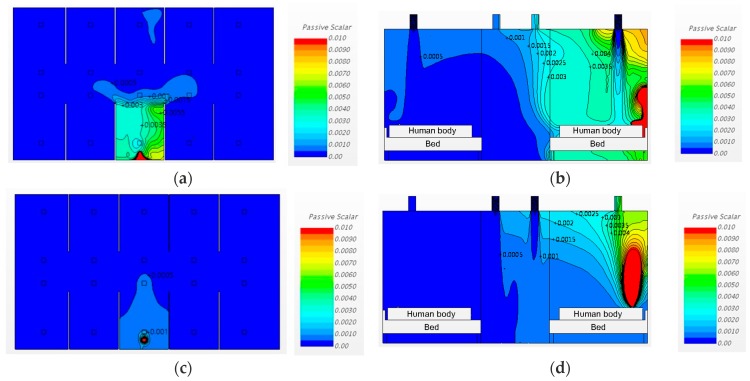
Airflow patterns and pathogen concentrations for Cases 7 and 8: (**a**) Case 7, plan view (h = 1.0 m); (**b**) Case 7, sectional view; (**c**) Case 8, plan view (h = 1.0 m); (**d**) Case 8, sectional view.

Figure 11 shows the analysis result of the cases considering the conditions of installing partitions between the beds, changing the ventilation system inlet/outlet locations, and increasing the ventilation rate to 6 ACH.

The simulated results of Cases 7 and 8 demonstrate that the increase in airborne pathogen concentration occurred mainly at X3. The increased ventilation ensures a solution for airborne infection prevention. The effects of installing partitions at a ventilation rate of 6 ACH varied depending on the ventilation system’s inlet and outlet locations. Locating the inlets above the patients’ respiratory organs still generated turbulence and increased the airborne pathogen concentration at the adjacent beds. The simulation results of Case 8 clearly show that the increased ventilation rate of 6 ACH and partition installation effectively reduced the airborne pathogen dispersion. However, the large increase in the pathogen concentration at the pathogen generation bed must be mitigated.

## 4. Discussion

The study focused on the effects of some potential solutions for preventing the spread of airborne pathogens in EDs: Changing the ventilation inlet/outlet locations, installing partitions, and increasing the ventilation rate.

In terms of ventilating the ED, supplying the air from the center (Region B) and exhausting over the patients’ respiratory systems (Region A) helped prevent dispersion of the airborne pathogen when partitions were not present. Changing the ventilation inlet and outlet locations in conditions of low ventilation rate and partition installation could cause the undesirable dispersion of the airborne pathogen (Case 6). Installing the outlet of the ventilation system over the patients’ respiratory systems was found to greatly increase the pathogen concentrations around the airborne pathogen source. Yang et al. (2017) analyzed by CFD, it was said that setting the parallel bed layout and the exhaust port near the patient’s head will lower the probability of air infection in the isolating room [20]. Hospital managers should be considered the placement of beds and the direction of airflow to reduce airborne infections. Ordinary breathing generates lots of bio-aerosol (1 μm or smaller) that can be transferred via air [21,22,23]. Thus, it is concluded that switching the ventilation inlet/outlet locations presents a high risk of increasing the probability of infection for medical staff providing medical treatments, such as endotracheal intubation [24,25] and nebulizing [26], to patients.

Installing partitions between the beds generally offered the advantage of reducing the average concentrations of pathogens in the ED (Cases 5, 7, and 8). However, it also had the shortcoming of increasing the pathogen concentration at the beds opposite and adjacent to the pathogen-source bed. These results suggest that patients who are at high risk of airborne infections may be able to provide appropriate patient placement in the ED. Physical blocks primarily prevent the spread of airborne pathogen between the emergency beds [9]. According to the CFD analysis, hospital curtains were presented in a simple and effective way for the spread of infection [27]. Thus, the airborne pathogens tend to accumulate around the source. Increased ventilation should, therefore, accompany partition installation in the ED. It should be 6 ACH recommended by WHO rather than Korean ventilation’s criteria. Increasing ventilation rate requires additional HVAC system installation and surplus energy consumption. Natural ventilation or hybrid ventilation method may be implemented to reduce energy consumption. However, natural ventilation has limitation or disadvantage regarding airflow control, heat recovery, outdoor contaminants incoming [28], difficulty in securing sufficient and consistent ventilation rate by season changing [29,30]. To reduce energy consumption, partial fan assisted natural ventilation with appropriate filtering system, dedicated outdoor air system (intermediate season) and heat recovery system (heating season) in HVAC system can be good solutions.

Most hospitals use partitions, but each hospital has a different material. Future research of the effect of airborne infection of the partition is also necessary. Future research needs to investigate the influence of airborne infection depending on the material, installation structure and usage method of the partition.

The South Korean government tries to enhance the quality of ED facilities in major hospitals to prevent airborne pathogen infections. However, the Korean building code still does not require the performance enhancement of small hospital and ED building equipment with respect to airborne infection prevention. This study provides the basis for design guidelines for airborne-infection prevention for small hospitals and EDs. The limitation of this study did not take into account the various factors affecting the airflow, such as natural ventilations. Future research needs to consider the airflow around the ceiling diffuser and approach the multidisciplinary research on hospital facilities and built environment will be conducted.

## 5. Conclusions

Our research investigated the effect of various architectural features in EDs on indoor pathogen concentration distribution. The major variables considered were ventilation rate, installation of partition walls between beds, and changing ventilation inlet/outlet locations. The overall analysis is summarized as follows.

(1). The most effective method for controlling airborne pathogen dispersion is increasing the ventilation rate.
(2). Changing the ventilation inlet/outlet locations generally results in good prevention of airborne pathogen dispersion. However, it can also cause undesirable airborne pathogen dispersion in conditions with low ventilation rates and partitions (Case 6).
(3). Installing partitions could contribute to decreasing the average airborne pathogen concentration (Cases 5, 7 and 8). However, it was also observed that the partitions could increase the pathogen concentrations in the beds opposite and adjacent to the pathogen source. Increasing the ventilation rate can enhance the effect of installing partitions.
(4). In the analysis, the most effective method for pathogen control was to use all the methods studied: Increasing the ventilation rate, installing partitions, and positioning the ventilation system outlets over the patients’ respiratory organs (Region A).

Further research will be conducted to improve architectural techniques for preventing the dispersion of airborne pathogens. Our research only analyzed the concentrations of the airborne pathogen; therefore, it has the limitation of not indicating the real probability of infection. In our future research, an analysis of the probability of infection by the airborne pathogen will be included.

## Figures and Tables

**Table 1:** Boundary conditions in the computational fluid dynamics (CFD) simulations.

Types	Base Model & Type 1
Physical conditions	Steady state flow: Segregated flow energy: Segregated fluid temperature turbulence: Realizable k-ε turbulence model gas: Air, ideal gas, incompressible gravity: −9.81 m/s^2^ Solver: Steady state
Meshing conditions	type: Polyhedral mesh prism layer thickness: 33% of the base (6 layers)
Inlet conditions 1 (diffuser)	velocity inlet (size: 0.15 m × 0.15 m) turbulence intensity: 2.5%, turbulent viscosity ratio: 10 3 ACH conditions: 0.62 m/s 6 ACH: conditions: 1.24 m/s
Outlet conditions (diffuser)	split ratio outlet (size: 0.15 m × 0.15 m)
Inlet conditions 2 (Exhalation with airborne pathogen)	velocity inlet (size: 0.04 m × 0.02 m) turbulence intensity: 1%, turbulent viscosity ratio: 10 passive scalar concentration: 1
Ventilation rate	3 ACH (base) or 6 ACH (type 1)
Thermal	inlet air flow: 23 °C, wall: 22 °C human body: 31 °C, exhalation: 36.5 °C
Convergence criteria	<10^−4^

**Table 2:** Meshing conditions.

Models	Number of Cells (Volume Mesh)
Base model	2,405,265
Type 1	2,691,618

**Table 3:** Simulation cases.

Model	Case #	Partition Condition	Ventilation Rate	Diffuser Location
Base model	Case 1	Without partition	3 ACH	Inlet (Region A) Outlet (Region B)
	Case 2			Inlet (Region B) Outlet (Region A)
	Case 3		6 ACH	Inlet (Region A) Outlet (Region B)
	Case 4			Inlet (Region B) Outlet (Region A)
Type 1	Case 5	With partition	3 ACH	Inlet (Region A) Outlet (Region B)
	Case 6			Inlet (Region B) Outlet (Region A)
	Case 7		6 ACH	Inlet (Region A) Outlet (Region B)
	Case 8			Inlet (Region B) Outlet (Region A)

## Appendix A

**Table A1:** Estimated number of airborne pathogens.

Location	Estimated Number of Airborne Pathogens at Each Location (Particles)								Notes
	Case 1	Case 2	Case 3	Case 4	Case 5	Case 6	Case 7	Case 8	
X1-Y1	1.83	1.75	0.80	0.47	0.06	0.24	0.02	0.04	patients nose
X1-Y2	1.64	1.06	0.71	0.45	0.08	0.27	0.02	0.05	
X1-Y3	1.26	0.71	0.47	0.38	0.09	0.29	0.02	0.06	
X1-Y4	0.76	0.64	0.26	0.28	0.09	0.26	0.02	0.05	
X1-Y5	0.70	0.57	0.25	0.28	0.06	0.23	0.02	0.04	patients nose
X2-Y1	1.51	1.28	0.80	0.47	0.26	0.57	0.18	0.15	patients nose
X2-Y2	1.49	1.15	0.70	0.54	0.36	0.58	0.19	0.16	
X2-Y3	1.14	0.67	0.43	0.40	0.32	0.85	0.35	0.20	
X2-Y4	0.79	0.60	0.42	0.27	0.41	0.67	0.16	0.15	
X2-Y5	0.56	0.56	0.34	0.23	0.33	0.67	0.16	0.13	patients nose
X3-Y1	5.61	7.16	4.68	4.79	3.28	62.11	3.77	66.51	pathogen source
X3-Y2	1.68	1.28	1.24	0.87	2.53	1.40	3.08	0.60	
X3-Y3	1.03	0.56	0.64	0.30	0.71	1.38	0.40	0.43	
X3-Y4	0.75	0.48	0.34	0.24	0.85	1.01	0.42	0.27	
X3-Y5	0.62	0.48	0.29	0.23	0.59	1.01	0.46	0.26	patients nose
X4-Y1	1.44	1.37	0.73	0.46	0.28	0.56	0.38	0.15	patients nose
X4-Y2	1.41	1.00	0.64	0.54	0.37	0.58	0.41	0.18	
X4-Y3	1.13	0.61	0.46	0.30	0.34	0.85	0.55	0.23	
X4-Y4	0.74	0.55	0.37	0.26	0.40	0.62	0.27	0.15	
X4-Y5	0.58	0.55	0.30	0.23	0.32	0.62	0.26	0.14	patients nose
X5-Y1	1.58	1.58	0.89	0.50	0.05	0.25	0.03	0.05	patients nose
X5-Y2	1.52	0.94	0.73	0.47	0.09	0.27	0.04	0.05	
X5-Y3	1.19	0.68	0.47	0.40	0.09	0.35	0.05	0.08	
X5-Y4	0.65	0.60	0.26	0.28	0.09	0.26	0.03	0.05	
X5-Y5	0.62	0.56	0.24	0.26	0.05	0.24	0.03	0.04	patients nose

**Table A2:** Estimated airborne infection risk.

Location	Estimated Airborne Infection Risk at Each Location (%)								Notes
	Case 1	Case 2	Case 3	Case 4	Case 5	Case 6	Case 7	Case 8	
X1-Y1	99%	98%	84%	66%	13%	42%	5%	9%	patients nose
X1-Y2	98%	91%	81%	65%	17%	46%	5%	11%	
X1-Y3	95%	81%	66%	58%	19%	49%	5%	13%	
X1-Y4	83%	77%	45%	48%	19%	45%	5%	11%	
X1-Y5	80%	73%	44%	48%	13%	41%	5%	9%	patients nose
X2-Y1	97%	95%	84%	66%	45%	73%	34%	29%	patients nose
X2-Y2	97%	93%	80%	71%	56%	74%	35%	31%	
X2-Y3	93%	79%	63%	60%	52%	86%	55%	37%	
X2-Y4	84%	75%	62%	46%	61%	79%	31%	29%	
X2-Y5	72%	72%	54%	41%	53%	79%	31%	26%	patients nose
X3-Y1	100%	100%	100%	100%	100%	100%	100%	100%	pathogen source
X3-Y2	98%	95%	94%	87%	100%	96%	100%	75%	
X3-Y3	91%	72%	77%	50%	81%	96%	60%	63%	
X3-Y4	82%	67%	54%	42%	86%	90%	62%	46%	
X3-Y5	76%	67%	49%	41%	74%	90%	65%	45%	patients nose
X4-Y1	96%	96%	81%	65%	48%	72%	58%	29%	patients nose
X4-Y2	96%	90%	77%	71%	57%	74%	61%	34%	
X4-Y3	93%	75%	65%	50%	54%	86%	72%	41%	
X4-Y4	82%	72%	57%	45%	60%	76%	46%	29%	
X4-Y5	74%	72%	50%	41%	52%	76%	45%	28%	patients nose
X5-Y1	97%	97%	87%	68%	11%	44%	7%	11%	patients nose
X5-Y2	97%	89%	81%	66%	19%	46%	9%	11%	
X5-Y3	94%	79%	66%	60%	19%	55%	11%	17%	
X5-Y4	78%	75%	45%	48%	19%	45%	7%	11%	
X5-Y5	76%	72%	42%	45%	11%	42%	7%	9%	patients nose
